# A molecular signature for the prediction of recurrence in colorectal cancer

**DOI:** 10.1186/s12943-015-0296-2

**Published:** 2015-02-03

**Authors:** Lisha Wang, Xiaohan Shen, Zhimin Wang, Xiuying Xiao, Ping Wei, Qifeng Wang, Fei Ren, Yiqin Wang, Zebing Liu, Weiqi Sheng, Wei Huang, Xiaoyan Zhou, Xiang Du

**Affiliations:** Department of Pathology, Fudan University Shanghai Cancer Center, Shanghai, 200032 China; Department of Oncology, Shanghai Medical College, Fudan University, Shanghai, 200032 China; Institute of Pathology, Fudan University, Shanghai, 200032 China; Institutes of Biomedical Sciences, Fudan University, Shanghai, 200032 China; Department of Genetics, Shanghai-MOST Key Laboratory of Health and Disease Genomics, Chinese National Human Genome Center and Shanghai Industrial Technology Institute, Shanghai, 201203 China; Department of Oncology, Renji Hospital, School of Medicine, Shanghai Jiaotong University, Shanghai, 200127 China

**Keywords:** Molecular signature, Gene expression, Colorectal cancer, Recurrence

## Abstract

**Background:**

Several clinical and pathological factors have an impact on the prognosis of colorectal cancer (CRC), but they are not yet adequate for risk assessment. We aimed to identify a molecular signature that can reliably identify CRC patients at high risk for recurrence.

**Results:**

Two hundred eighty-one CRC samples (stage II/III) were included in this study. A two-step gene expression profiling study was conducted. First, gene expression measurements from 81 fresh frozen CRC samples were obtained using Affymetrix Human Genome U133 Plus 2.0 Arrays. Second, a focused gene expression assay, including prognostic genes and genes of interest from literature reviews, was performed using 200 fresh frozen samples and a Taqman low-density array (TLDA) analysis. An optimal 31-gene expression classifier for the prediction of recurrence among patients with stage II/III CRC was developed using logistic regression analysis. This gene expression signature classified 58.5% of patients as low-risk and 41.5% as high-risk (*P* < 0.001). The signature was the strongest independent prognostic factor in the multivariate analysis. The five-year relapse-free survival (RFS) rates for the low-risk patients and the high-risk patients were 88.5% and 41.3% (*P* < 0.001), respectively.

**Conclusion:**

We identified a 31-gene expression signature that is closely associated with the clinical outcome of stage II/III CRC patients.

## Introduction

Colorectal cancer (CRC) is the third most common type of cancer, with a worldwide annual incidence of over 1.2 million cases and a mortality rate of approximately 50% [1,2]. The growing awareness that CRC is a heterogeneous disease with respect to clinical behavior and prognosis translates into an urgent need for robust molecular subclassifiers in addition to the current parameters. To date, some clinical and pathologic features, such as intestinal perforation/obstruction, adjacent organ involvement (T4), high tumor grade, lymphatic/vascular invasion and inadequate sampling of lymph nodes, can identify a minority of CRC patients who are at a high risk of recurrence [3,4]. However, these prognostic factors are all relatively weak.

To address this issue, the prognostic potential of molecular markers, including chromosome and microsatellite instability (MSI) and the mutation status of the *KRAS* or *BRAF* genes, has been systematically investigated in CRC [5-8]. Thus far, only *KRAS* mutation analysis has been used in clinical practice as a predictive marker of the effect of *EGFR* antibodies in metastatic disease [5,9]. Analyses of other known critical CRC molecular markers are not currently recommended for screening or for prognostic prediction because they require further validation.

With the recent advent of microarray technology, risk assessment for CRC has been improved by the use of gene expression profiling. DNA microarray technology can measure thousands of mRNA transcripts at once and may be able to describe the complex biology of a tumor more accurately than single markers [10,11]. In the current study, we used gene expression analysis data from recurrent and non-recurrent patients with CRC to identify differentially expressed probes. To further validate gene expression, we selected 48 genes that could be assayed using a TaqMan low-density array (TLDA), a real time quantitative PCR (RT-qPCR) based technology, using fresh frozen CRC tissues.

## Patients and methods

### Patients and tumor samples

Samples were prospectively collected between 2007 and 2009 at Fudan University Shanghai Cancer Center. The inclusion criteria were as follows: primary sporadic colorectal adenocarcinoma (excluding familial adenomatous polyposis (FAP) and hereditary nonpolyposis colorectal cancer (HNPCC)), 18 to 75 years of age, no preoperative chemotherapy and radiotherapy, and similar postoperative chemotherapy regimens. The patients were staged according to the American Joint Committee on Cancer/International Union against Cancer (AJCC/UICC) TNM staging system- seventh edition (2010). Histologic grading (differentiation) was based on the WHO classification of tumors of the digestive system-fourth edition (2010). This study was approved by the Ethical Committee of our Cancer Center, and written informed consent was obtained from each patient.

### Microarray gene expression profiling

Tumor tissues were taken from 81 patients with CRC, rapidly frozen in RNAlater, and stored at −80°C until processing. All samples were visually inspected by two pathologists, who confirmed the presence of tumor cells (≥70%) in all samples. RNA was isolated using Trizol (Life Technologies, Carlsbad, California, USA) and purified using RNeasy MinElute Cleanup Kit (QIAGEN, Hilden, Germany) as recommended by the manufacturers. Quantity and quality measurements were carried out using a NanoVue™ Plus Spectrophotometer (GE, London, UK) and an Agilent 2100 Bioanalyzer (Agilent Technologies, Santa Clara, California, USA). Gene expression profiles were determined using Affymetrix HG-U133 Plus 2.0 GeneChips according to the recommendations of the manufacturer.

### TaqMan low density array (TLDA)

Two hundred fresh frozen CRC samples were used for TLDA analysis. Pre-designed TaqMan probe and primer sets for target genes were chosen from an online catalog (Applied Biosystems). Once selected, the primer sets were factory loaded into the 384 wells of TLDA cards. Each TLDA card in this study was configured into 8 identical 48-gene sets (2 samples in duplicate). In our study, 48 genes were chosen based on gene expression profiling analysis and literature reviews [3,12-22] (Table 1). Each set contained *GAPDH*, *UBC*, and *18S*, all of which were used as reference genes for normalization [23,24]. These genes were demonstrated to be expressed in colorectal tissues with little variability. The geometric mean was used for the calculation of the expression of target genes.

**Table 1:** **Gene expression assays used for configuring the Taqman low-density array card**

**Probe sets/Ref. sequences**	**Gene symbol**	**Assay IDs**	**Probe sets/Ref. sequences**	**Gene symbol**	**Assay IDs**
1554997_a_at	*PTGS2*	Hs01573477_g1	232278_s_at	*DEPDC1*	AJCSVBJ
1558135_at	*TAF11*	Hs01051508_g1	232315_at	*LOC400713*	AJ89JTA
1562921_at	*GENE3*	AJD1THR	232684_at	*LOC253264*	AJGJPT7
200632_s_at	*NDRG1*	Hs00608390_m1	234768_at	*GENE28*	Hs01078763_m1
203001_s_at	*STMN2*	Hs00975902_m1	235229_at	*GENE29*	AJMSGPB
203889_at	*SCG5*	Hs02559426_s1	238531_x_at	*GENE30*	AJ5IPAM
204886_at	*PLK4*	Hs00975273_m1	238629_x_at	*GENE31*	AJN1EVJ
204932_at	*GENE8*	AJ6RNGU	241607_at	*LOC730102*	AJBJW5B
204933_s_at	*TNFRSF11B*	Hs00900360_m1	59437_at	*C9orf116*	Hs00376168_m1
205828_at	*MMP3*	Hs00968308_m1	NM_001025366	*VEGFA*	Hs00900055_m1
205890_s_at	*UBD*	Hs00197374_m1	NM_000610	*CD44*	Hs01075861_m1
207808_s_at	*PROS1*	Hs00165590_m1	NM_004530	*MMP2*	Hs01548727_m1
211653_x_at	*AKR1C2*	AJ70LM2	NM_003254	*TIMP1*	Hs00171558_m1
212315_s_at	*NUP210*	Hs01090284_m1	NM_004994	*MMP9*	Hs00234579_m1
215039_at	*LOC339524*	AJKAKCV	NM_002046	*GAPDH*	Hs02758991_g1
219054_at	*C5orf23*	AJHSN0F	NM_004360	*CDH1*	Hs01023894_m1
219148_at	*PBK*	Hs00218544_m1	NM_005429	*VEGFC*	Hs00153458_m1
220295_x_at	*GENE18*	AJFARNZ	NM_003345	*UBC*	Hs01871556_s1
221703_at	*BRIP1*	Hs00908148_m1	NM_001098210	*CTNNB1*	Hs00355049_m1
226211_at	*MEG3*	AJLJII3	NM_015675	*GADD45B*	Hs04188837_g1
226661_at	*CDCA2*	Hs00401022_m1	NM_002466	*MYBL2*	Hs00942543_m1
228877_at	*RGL3*	Hs01004328_g1	NM_000876	*IGF2R*	Hs00974474_m1
229331_at	*SPATA18*	Hs01111872_g1	NM_000269	*NME1*	Hs02621161_s1
230135_at	*GENE24*	AID1TYD			

Total RNA was reverse-transcribed using the High-Capacity cDNA Reverse Transcription Kit using reaction volumes of 10 μL (Applied Biosystems, Foster City, CA). A total of 100 μL reaction mixture containing 9 μL of cDNA template (corresponding to 100 ng of mRNA) and 50 μL of 2× TaqMan Universal PCR Master Mix (Applied Biosystems, Foster City, CA, USA) was added to each line of TLDA after vortex and brief centrifugation. One microliter of the reaction mixture, which contained 1 ng of mRNA, was transferred to each reaction cell. The TLDA plates were sealed with a TLDA sealer before centrifugation in a Thermo Scientific Sorvall ST40 centrifuge. PCR amplification was carried out in the micro-fluidic card sample block of an ABI Prism® 7900HT sequence detection system (Applied Biosystems, Foster City, CA, USA). The amplification protocol was as follows: 10 min at 94.5°C (activation), 40 cycles of denaturation at 95°C for 15 s, and annealing and extension at 59.7°C for 1 min.

### Statistics

The gene expression data were filtered and normalized using Expression Console™ 1.2.0.20 software to remove systematic technical variation before further analysis. Raw data had been deposited in the Gene Expression Omnibus (GEO) database and were accessible through the accession number GSE64857. To reduce variation between individual microarrays, the intensity values for the samples in each microarray were rescaled by means of a quantile normalization method. Each intensity value was log-transformed to a base-2 scale. The intensity value was coded as 1 for expression levels ranked at or below the 25th percentile of total gene expression, 2 for levels above the 25th percentile and at or below the 50th percentile, 3 for levels above the 50th percentile and at or below the 75th percentile, and 4 for levels above the 75th percentile [25].

The threshold cycle (Ct) generated from TLDA analysis was automatically determined using the SDS2.2 software package. The relative quantities (RQ) were determined using the equation: RQ = 2^-ΔΔCt.^ To reduce variation between TLDA analyses, the Ct values for samples on each TLDA card were also rescaled by means of a quantile normalization method. Each intensity value was log-transformed to a base-2 scale. The expression intensities were ranked as 1, 2, 3, and 4. A binary logistic regression model was performed to discriminate between the recurrent group and the non-recurrent group using a stepwise backward conditional procedure, and probability was set at 0.05 for entry and 0.10 for removal. A patient was classified into the high risk group if the value was greater than 0.5 or into the low risk group if the value was less than 0.5.

Further statistical analyses were performed using SPSS 16.0 software, including independent samples t tests, χ^2^ tests, univariate and multivariate Cox proportional hazards analyses (estimation of HR and the corresponding 95% confidence intervals), and Kaplan-Meier survival analysis (differences were assessed by log-rank statistics). For survival analyses, the end-point was relapse-free survival (RFS), which was defined as the probability that patients remain free of recurrence (local/regional or metastatic) as the first event. A two-sided *P*-value of ≤ 0.05 was considered to be significant.

## Results

### Clinicopathologic characteristics

**Figure 1:**
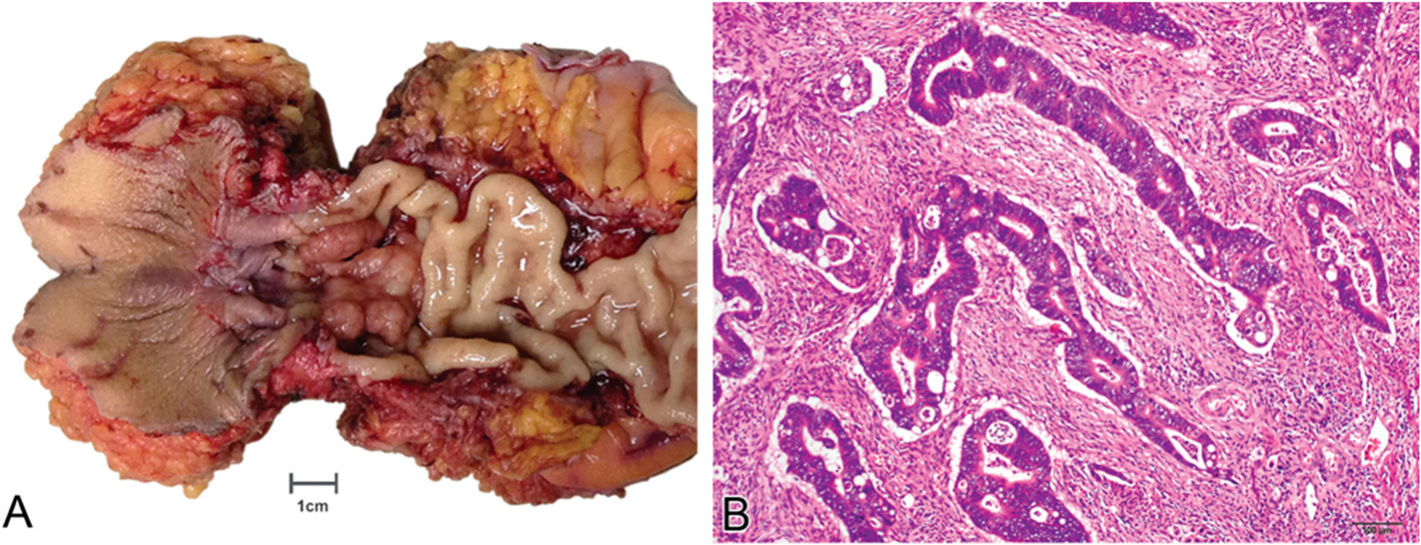
**Gross appearance and microscopic findings of colorectal cancer. (A)**, Gross morphology of an opened Miles’ abdominoperineal resection specimen containing an exophytic mass. The bowel lumen can become progressively narrowed and constricted. **(B)**, Representative images of a moderately differentiated colorectal adenocarcinoma (H&E stains, ×100). The tumor cells are arranged in glandular patterns, with a prominent desmoplastic stromal response.

A total of 281 fresh frozen CRC specimens were included in the study (Figure 1), and 81 of these tumors were used for microarray gene expression analysis. Of these 81 patients, 47 were male and 34 were female. The patients’ ages ranged from 29 to 75 years (median 56.3 years). The mean follow-up time was 48 months (range 38–68 months). Twenty-six patients had relapsed or died at the time of last follow-up. Six patients were lost to follow-up. The metastatic sites included the liver (n = 9), lungs (n =4), abdominal sites (n = 2), and other organs.

Two hundred tumors were used for TLDA analysis. These tumors were obtained from 117 male patients and 83 female patients. The ages of the patients ranged from 32 to 75 years (median 57.4 years). The mean follow-up time was 48 months (range 36–68 months). Fifty-five patients relapsed or died at the time of last follow-up. The metastatic sites included the liver (n = 17), lungs (n =9), abdominal sites (n = 6), bones (n = 3), and other organs.

### Gene expression analysis

**Figure 2:**
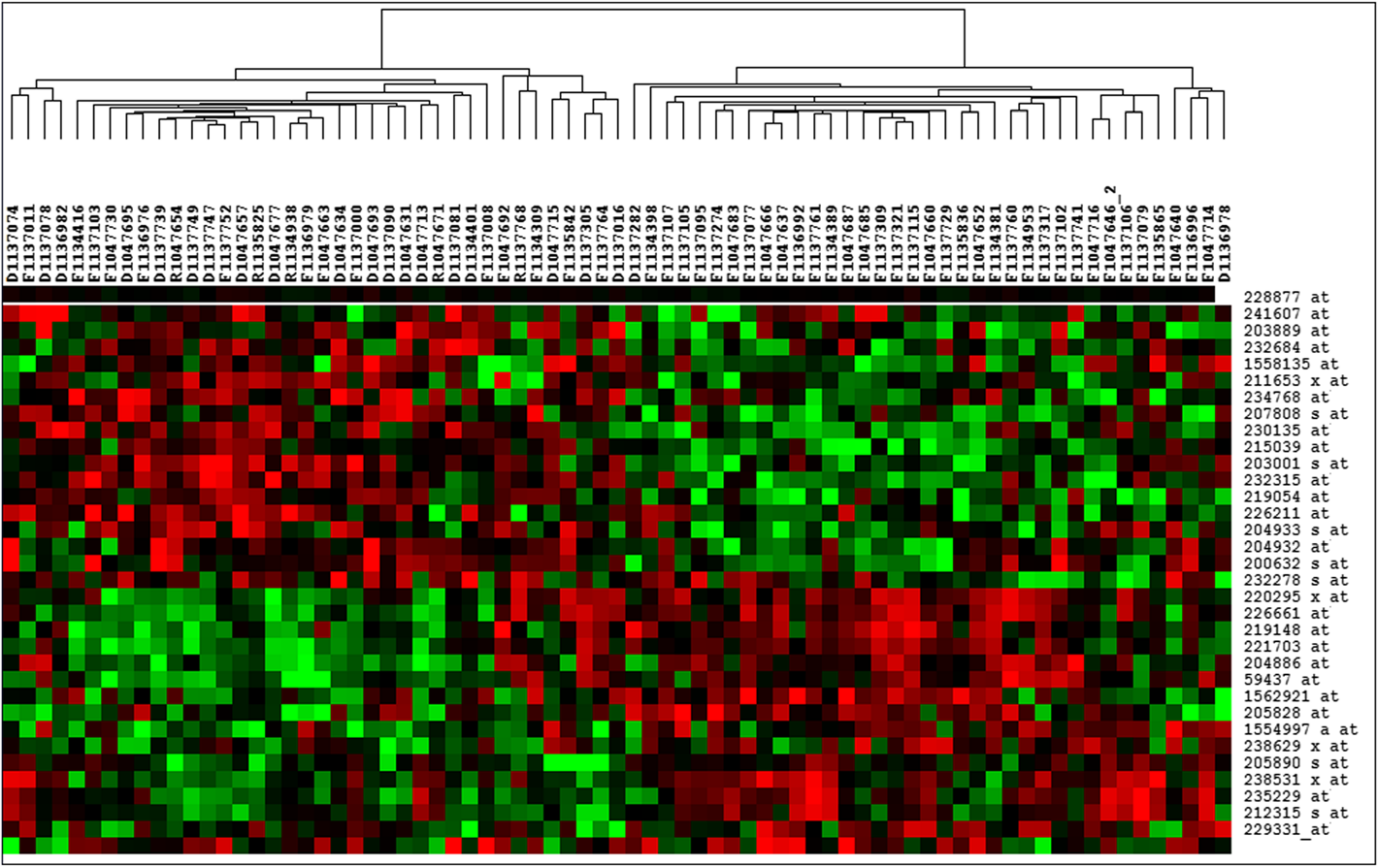
**Hierarchical cluster analysis of colorectal cancer (26 recurrent patients vs. 49 non-recurrent patients).** The data are presented here in matrix format, in which the rows represent individual genes (31 probes sets) and the columns represent each tissue from CRC patients (“D” = death; “R” = relapse; “F” = survival). The red and green colors reflect high and low expression levels, respectively.

Genome-wide expression data were used to identify genes that correlate with patient prognosis. Thirty-three probe sets were selected according to the following criteria: (1) fold change ≥1.5 or ≤0.67; (2) raw gene expression signal levels above 100 in 60% of the samples; (3) *P* ≤ 0.01 (Figure 2). The 33-gene panel was calculated as follows: y = C+(B1×1…B33×33), P = EXP(y)/(1 + EXP(y)), where C represents the constant (C = −160.677788) and B represents the weight coefficient for each gene. The expression levels of these 33 genes correlated with relapse. The probe sets are summarized in Table 1. The sensitivity and specificity of this panel are 92.31% and 71.43%, respectively. The Hosmer-Lemeshow (HL) test showed good fit of the model (*P* = 1). As a continuous variable, the genetic signature classified patients into two groups using 0.5 as a cutoff; group 1 with *P* < 0.5 represented the non-recurrent patients, and group 2 with *P* > 0.5 represented the recurrent patients.

### TaqMan low density array analysis

**Figure 3:**
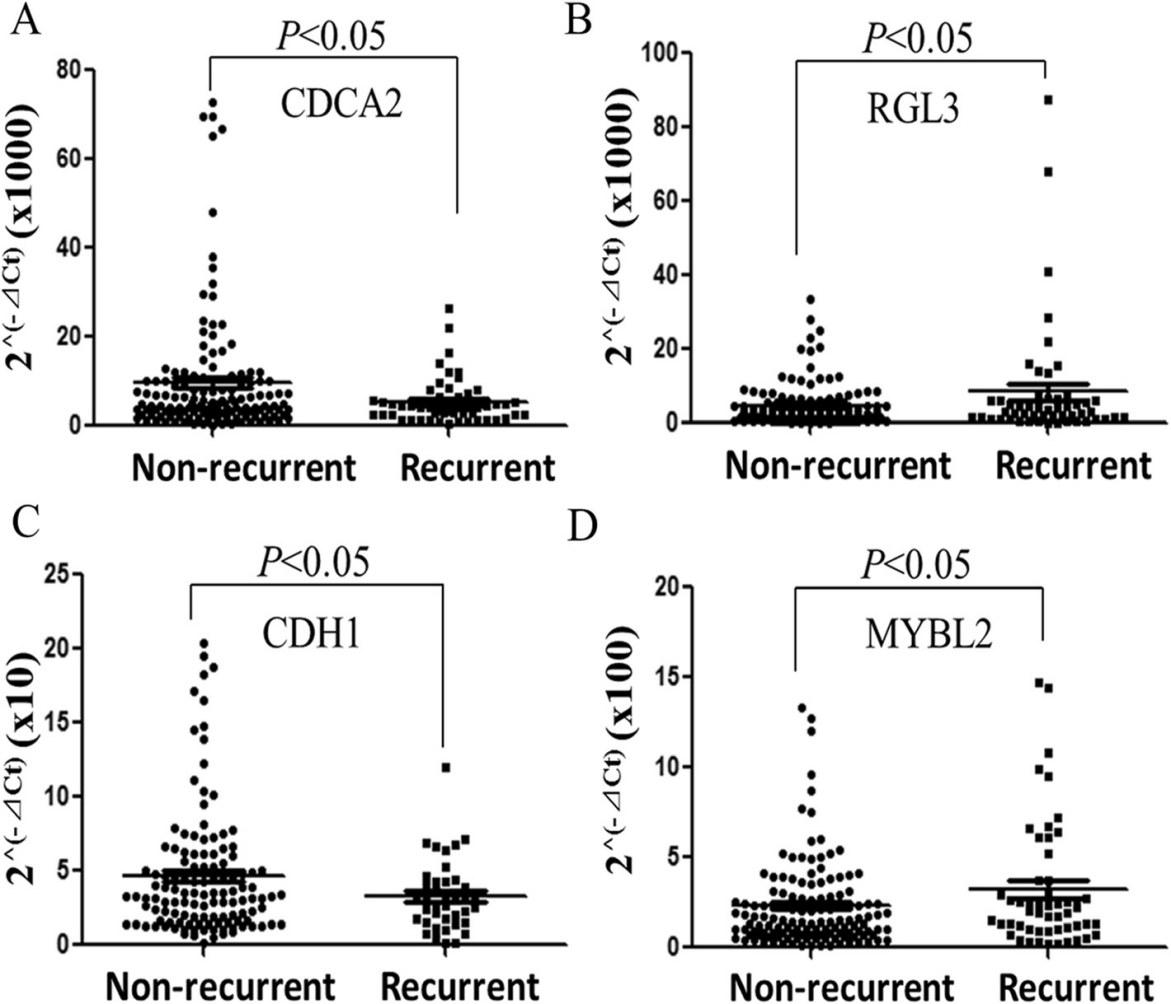
**mRNA levels of representative genes in the recurrent group and the non-recurrent group. (A)**, The level of *CDCA2* mRNA expression was significantly increased in the non-recurrent group compared to the recurrent group. **(B)**, The level of *RGL3* mRNA expression was significantly increased in the recurrent group. **(C)**, The level of *CDH1* was significantly increased in the non-recurrent group. **(D)**, The level of *MYBL2* was significantly increased in the recurrent group.

The results of microarray analysis prompted us to implement a TLDA assay with the differentially expressed probes. In total, 48 genes were chosen for the TLDA assay, including 33 candidate genes that are differentially expressed based on the microarray analysis, 12 relative genes selected from a literature review, and 3 reference genes. Forty-six genes (95.8%, 46/48) were successfully amplified by TLDA. Seventeen genes (*PTGS2*, *NUP210*, *PBK*, *GENE18*, *MEG3*, *CDCA2*, *RGL3*, *DEPDC1*, *LOC400713*, *GENE29*, *MMP2*, *TIMP1*, *MMP9*, *CDH1*, *GADD45B*, *MYBL2*, and *IGF2R*) were differentially expressed in the recurrent group and the non-recurrent group based on the TLDA results (*t* test, *P* < 0.05). Eleven genes were significantly down-regulated in the recurrent group, while 6 genes were significantly up-regulated. Among these 17 genes (17-gene panel), 10 genes (10-gene panel) were identified from our microarray analysis, and 7 genes (7-gene panel) that were identified by other studies were also confirmed. The fold changes of these genes are summarized in Table 2. The mRNA levels of the target genes were calculated as 2^^ (−ΔCt)^. Figure 3 shows the expression of *CDCA2*, *RGL3*, *CDH1*, and *MYBL2* in the recurrent group and the non-recurrent group.

**Table 2:** **mRNA expression in the recurrent group and the non-recurrent group as assayed using the Taqman low-density array assay**

**Gene symbol**	Fold change*	***P*** **value**	**Gene symbol**	Fold change*	***P*** **value**
*PTGS2*	0.485	**0.011**	*GENE24*	0.835	>0.05
*TAF11*	1.048	>0.05	*DEPDC1*	0.323	**0.0001**
*GENE3*	1.034	>0.05	*LOC400713*	2.280	**0.0419**
*NDRG1*	1.114	>0.05	*LOC253264*	1.436	>0.05
*STMN2*	0.994	>0.05	*GENE28*	1.049	>0.05
*SCG5*	0.938	>0.05	*GENE29*	0.346	**0.006**
*PLK4*	1.166	>0.05	*GENE30*	0.836	>0.05
*GENE8*	N/A	N/A	*GENE31*	N/A	>0.05
*TNFRSF11B*	1.049	>0.05	*LOC730102*	1.159	>0.05
*MMP3*	1.102	>0.05	*C9orf116*	1.003	>0.05
*UBD*	1.005	>0.05	*VEGFA*	1.228	>0.05
*PROS1*	0.774	>0.05	*CD44*	1.031	>0.05
*AKR1C2*	1.383	>0.05	*MMP2*	0.654	**0.0216**
*NUP210*	0.655	**0.0224**	*TIMP1*	2.153	**0.0199**
*LOC339524*	1.019	>0.05	*MMP9*	0.522	**0.0018**
*C5orf23*	0.691	>0.05	*CDH1*	0.712	**0.0071**
*PBK*	0.539	**0.0028**	*VEGFC*	1.013	>0.05
*GENE18*	0.617	**0.0317**	*CTNNB1*	1.007	>0.05
*BRIP1*	1.052	>0.05	*GADD45B*	1.881	**0.041**
*MEG3*	2.236	**0.0436**	*MYBL2*	1.398	**0.041**
*CDCA2*	0.556	**0.0011**	*IGF2R*	0.703	**0.0278**
*RGL3*	1.903	**0.0435**	*NME1*	1.174	>0.05
*SPATA18*	0.754	>0.05			

*: fold change, the ratio of gene expression in the recurrent group and the non-recurrent group. Bold text denotes *P*<0.05.

### The 31-gene signature and survival

**Figure 4:**
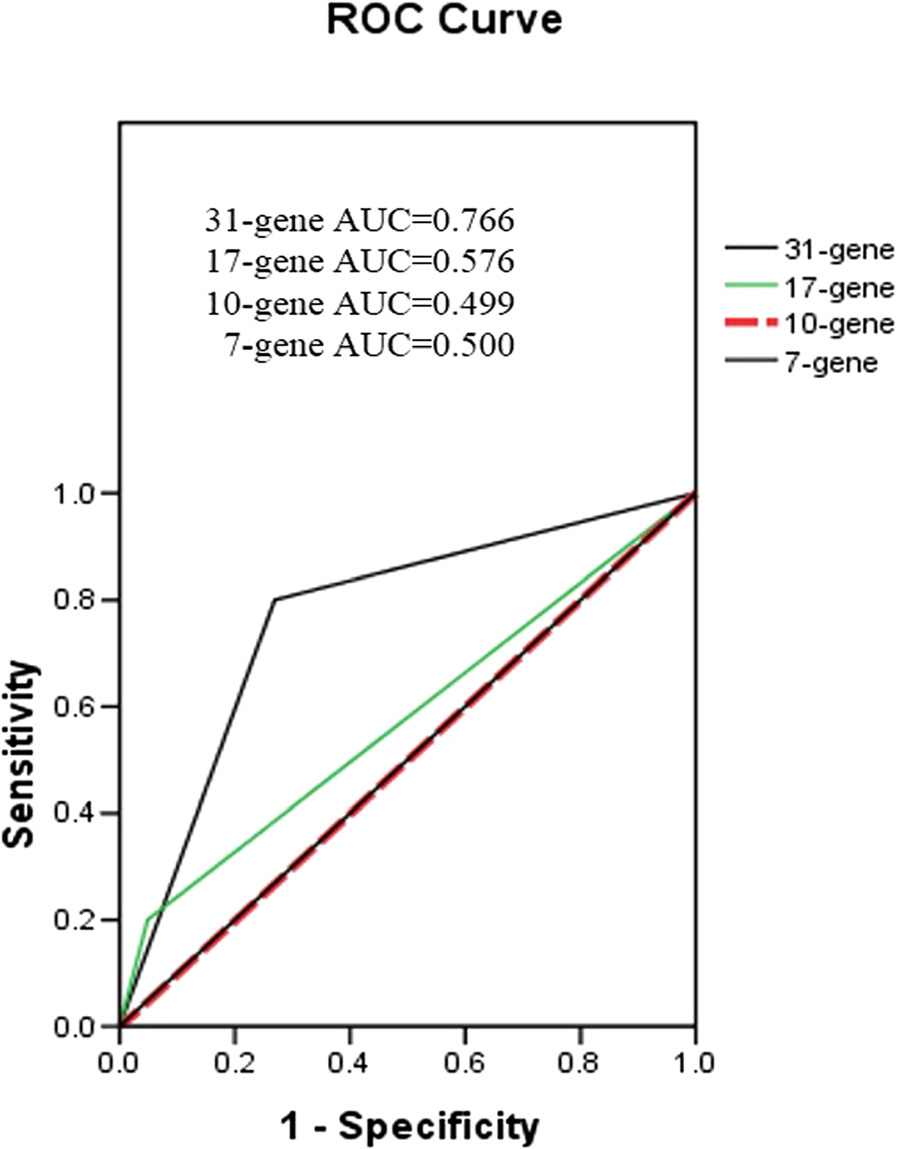
**Receiver operator characteristic (ROC) curve analyses of the 31-gene, 17-gene, 10-gene, and 7-gene panel.** Sensitivity was plotted on the y-axis, and 1-specificity was plotted on the x-axis. The area under the curve (AUC) was 0.766, 0.576, 0.499, and 0.500, respectively.

As not all of the genes amplified in the TLDA assay, a logistic regression analysis was performed in two steps to avoid the influence of missing values: 79 samples with full TLDA PCR data were first used to generate the formula; then all 200 samples, including 121 samples with partial missing data, were imported and entered into the formula for analysis. Among the 200 patients, 55 were recurrent, and 145 were non-recurrent. A 31-gene panel was developed using logistic regression analysis, each with a new weight coefficient. To compare the prediction performance of the 31-gene, 17-gene, 10-gene, and 7-gene panels, receiver operator characteristic (ROC) curves were drawn, and the area under the curve (AUC) was calculated for each ROC curve based on the standard leave-one-out procedure. The AUC demonstrated that the predicting performance of the 31-gene panel was the best one (0.766 vs 0.576 vs 0.499 vs 0.500, Figure 4). The use of this algorithm resulted in the separation of two groups, the recurrent group and the non-recurrent group. One hundred seventeen out of 200 patients (58.5%) were predicted to be non-recurrent patients (low risk); among these, 106 were verified as non-recurrent. Eighty-three out of 200 patients (41.5%) were predicted to be recurrent patients (high risk); among these, 44 were observed to be recurrent (*P* < 0.001). The positive predictive value, sensitivity and specificity of this set were 90.6%, 73.1% and 80.0%, respectively.

**Figure 5:**
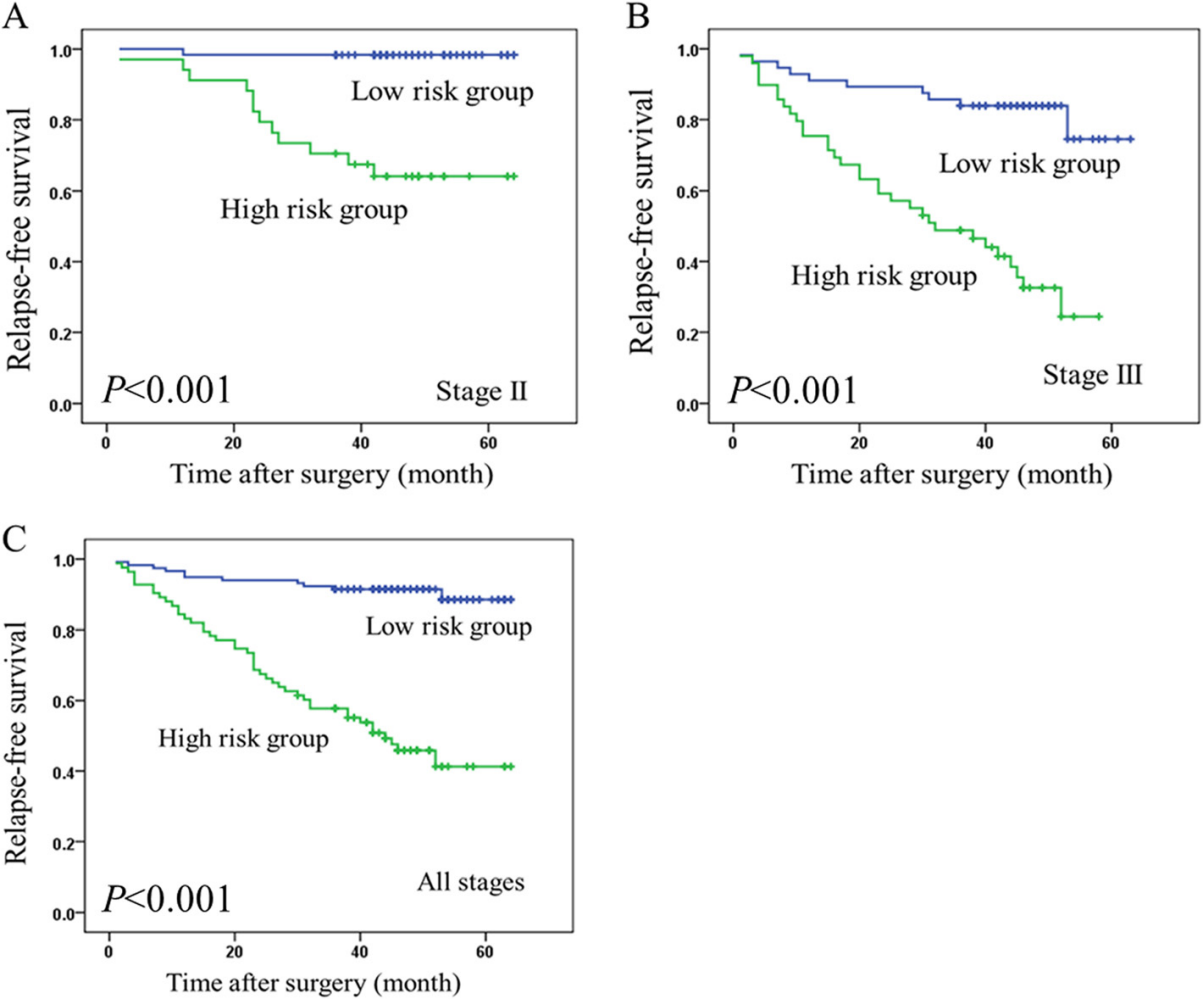
**Kaplan-Meier survival curves illustrating relapse-free survival among the recurrent patients and non-recurrent patients. (A)**, Stage II, n = 95; the 5-year relapse-free survival rate (RFS) for the low-risk patients was 98.4%, and the 5-year RFS of the high-risk patients was 64.1%. **(B)**, Stage III, n = 105; the 5-year RFS for low-risk patients was 74.6%, and the 5-year RFS for the high-risk patients was 24.4%. **(C)**, All stages, n = 200; the 5-year RFS for the low-risk patients was 88.5%, and the 5-year RFS for the high-risk patients was 41.3%.

Kaplan-Meier survival curves and the log-rank test were used to compare the survival differences between the two groups. The predicted low-risk group had a longer RFS than the predicted high-risk group (5-year RFS 88.5% vs. 41.3%, *P* < 0.001) (Figure 5). When the signature was separately applied to patients with stage II and stage III disease, it correctly classified the low- and high-risk patients in both groups (stage II, 5-year RFS 98.4% vs. 64.1%, *P* < 0.001; stage III, 5-year RFS 74.6% vs. 24.4%, *P* < 0.001). The univariate and multivariate Cox regression analyses of the clinical parameters and the 31-gene prognostic signature in CRC samples are provided in Table 3. In the univariate Cox regression analysis, TNM stage, T stage, N stage, and the prognostic signature were the strongest predictors of RFS. In the multivariate analysis, only the prognostic signature was a strong independent prognostic factor.

**Table 3:** **Univariate and multivariate Cox regression analysis of clinical factors and the 31-gene prognostic signature in colorectal samples**

**Parameters**	**No. of patients**	**Univariate**		**Multivariate**	
		***P*** **value**	**HR (95% CI)**	***P*** **value**	**HR (95% CI)**
Gender		0.303	1.321 (0.778-2.243)	0.940	1.022 (0.571-1.830)
Male	117				
Female	83				
Age		0.889	0.963 (0.656-1.641)	0.161	0.662 (0.372-1.178)
<60	113				
> = 60	87				
Tumor size		0.323	1.323 (0.759-2.308)	0.450	1.260 (0.692-2.296)
<5	141				
> = 5	59				
Location		0.376	0.776 (0.442-1.362)	0.895	0.961 (0.533-1.734)
Colon	77				
Rectal	123				
Gross appearance		0.820	1.062 (0.631-1.788)	0.599	0.863 (0.499-1.493)
Exophytic	39				
Ulcerative	5				
Infiltrative	156				
Differentiation		0.440	1.269 (0.693-2.323)	0.546	1.227 (0.631-2.388)
High	6				
Moderate	153				
Low	41				
Stage		**<0.001**	3.568 (1.912-6.660)	0.701	1.243 (0.410-3.764)
II	95				
III	105				
T stage		**0.025**	1.727 (1.069-2.790)	0.074	1.524 (0.960-2.420)
T2	15				
T3	80				
T4	105				
N stage		**<0.001**	2.569 (1.801-3.665)	**0.024**	2.101 (1.104-3.997)
N0	96				
N1	75				
N2	29				
Prognostic signature		**<0.001**	7.445 (3.835-14.454)	**<0.001**	6.752 (3.347-13.622)
High risk	83				
Low risk	117				

Bold text denotes *P*<0.05.

## Discussion

CRC is a heterogeneous disease. Even in patients with similar pathological and clinical features, the outcome varies; some are cured, but the cancer recurs in others. Although official guidelines give suggestions for risk stratification, there are no clear recommendations for the administration of adjuvant chemotherapy [26,27]. The staging systems for CRC may have reached their limit of usefulness for predicting outcomes, but molecular methods add value. In the present study, we developed a 31-gene expression signature for the prediction of relapse among patients with stage II/III CRC based on the combined use of gene expression profiling and TLDA analysis. The identification of 31 genes that are closely associated with outcomes in patients with CRC has clinical implications. We propose that patients who have a higher risk of relapse could benefit from adjuvant therapy and that those who have lower risk could be spared what may be unnecessary treatment.

In recent years, microarray analyses for the identification of differences in gene expression patterns have increased our understanding of the molecular genetic events in CRC. Several studies have identified gene expression signatures with a prognostic impact for patients with stage II and III CRC [11,28-31]. In early studies, small sample sizes and the lack of validation of independent sample sets limited the statistical power of the conclusions drawn. However, recent publications have addressed these limitations, and promising gene expression signatures have been suggested; two promising prognostic tests based on the expression levels of different gene panels have been reported. ColoPrint measures the expression levels of 18 genes, whereas Oncotype DX includes 12 genes, consisting of 7 recurrence risk genes and 5 reference genes [3,32]. However, there are few data points from Chinese patients. In our previous study, an 18-gene signature (*CD36, DHRS13, DUSP2, FAM198B, FKBP5, GLT25D2, GZMB, IL1B, ITGAM, ITPRIPL2, MYBL1, NEAT1, NUDT16, P2RY10, PDE4D, PDZK1IP1, SH2D2A,* and *VSIG10*) was identified that could accurately differentiate between peripheral blood samples of CRC patients and controls. These results open an avenue for the diagnosis and early detection of CRC [33]. Additional data are needed to develop multi-gene signatures to predict prognoses of Chinese patients. Based on the microarray analysis, 33 candidate genes were selected, and algorithms were developed to identify groups of patients with high and low risk of recurrence.

In this study, we have shown that TLDA, a robust methodology based on RT-qPCR, is suitable for the validation of expression profiling of target genes. Hundreds of reactions can be performed simultaneously, enabling a large number of samples to be rapidly assessed [34,35]. This technology would be especially useful for identifying gene signatures with prognostic significance. Gallagher WM et al. [36] combined the use of DNA microarray and a tailored TLDA card and identified multiple molecular determinants of melanoma progression. In this study, we have developed a TLDA assay that includes 33 genes that are differentially expressed in the recurrent group and the non-recurrent group and 12 relative genes based on a review of the literature. Forty-six genes (95.8%, 46/48) were successfully amplified by TLDA. Seventeen genes (*PTGS2*, *NUP210*, *PBK*, *GENE18*, *MEG3*, *CDCA2*, *RGL3*, *DEPDC1*, *LOC400713*, *GENE29*, *MMP2*, *TIMP1*, *MMP9*, *CDH1*, *GADD45B*, *MYBL2*, and *IGF2R*) were differentially expressed in the recurrent group and the non-recurrent group (*P* < 0.05). Eleven genes were significantly down-regulated in the recurrent group, while 6 genes were significantly up-regulated in the recurrent group. A 31-gene molecular signature was ultimately developed using logistic regression analysis *(PTGS2, TAF11, GENE3, SCG5, PLK4, TNFRSF11B, MMP3, UBD, AKR1C2, NUP210, LOC339524, C5orf23, PBK, BRIP1, MEG3, CDCA2, RGL3, SPATA18, GENE24, LOC400713, LOC253264, CSPP1, GENE30, C9orf11, CD44,* and *MMP2)*. The predicted low-risk group had a longer RFS than the predicted high-risk group (5-year RFS 88.5% vs. 41.3%, *P* < 0.001). When the signature was separately applied to patients with stage II and stage III disease, it correctly classified the low- and high-risk patients in both groups. A range of molecular markers were observed to correlate with CRC prognosis. The signature was the strongest independent prognostic factor in the univariate and multivariate analyses.

Our strategy has led to the discovery of recurrence risk genes that can be confidently associated with clinical outcome and has confirmed the previously reported associations of several genes with CRC. *PTGS2*, or cyclooxygenase 2 (*COX2*), is the rate-limiting enzyme in the production of proinflammatory prostaglandins, which contribute to a pro-angiogenic microenvironment in tumors [37]. Several studies have demonstrated that *PTGS2* is overexpressed in various tumors, including CRC [37-39]. Treatment with selective *PTGS2* inhibitors results in reduced colorectal neoplasia risk [40]. However, the data demonstrating a relationship between *PTGS2* expression and patient survival remain inconclusive. Fux R et al. [41] demonstrated that *PTGS2* expression in tumor epithelial cells is unrelated to overall survival and to DFS. Tougeron D et al. [42] reported that aspirin intake is associated with a significant improvement in the survival of CRC patients whose tumors carried mutant but not wild-type copies of the phosphoinositide 3-kinase (*PI3KCA*) gene. In a study by Aziz A et al. [43], overexpression of *PTGS2* is associated with better recurrence-free and disease-specific survival in a large cohort of patients with carcinoma invading bladder muscle treated by cystectomy. Similarly, in our study, *PTGS2* expression was significantly up-regulated in the non-recurrent group compared with the recurrent group. We postulated that *PTGS2* overexpression is involved in the initiation stage of carcinogenesis, but after tumors reach a level of aggressiveness (such as in the stage II/III cancer cases of the present study), the maintenance of *PTGS2* expression may be associated with tumors having a better prognosis. The potential of *PTGS2* as a prognostic marker in CRC should be considered.

Selected genes of interest in the tumor signature, including *VEGFA*, *VEGFC*, *CD44*, *CDH1*, *MMP2*, *MMP9*, *TIMP1*, *CTNNB1*, *GADD45B*, *MYBL2*, *IGF2R*, and *NME1*, are known to be involved in angiogenesis, metastasis, and prognosis. Among these genes, *MMP2*, *TIMP1*, *MMP9*, *CDH1*, *GADD45B*, *MYBL2*, and *IGF2R* were differentially expressed in the recurrent group and the non-recurrent group. To date, the relationships between *MMPs/TIMP* expression and patient survival are controversial. Wong JC [17] reported that *MMP2* was consistently underexpressed in liver metastases compared to primary CRCs. Shorter time to distant metastasis and overall survival occurred in stage III rectal cancer lacking *MMP2*. Furthermore, the authors confirmed that *MMP2* inhibitors promoted cell invasion in CRC cell lines in vitro. However, Hilska M et al. [44] reported that high expression of *MMP2* in the malignant epithelium and the surrounding stroma was associated with reduced survival in colon cancer patients. A multivariate analysis showed that *TIMP3* was the only marker with an independent prognostic value. In our study, the expression of *MMP2* was down-regulated in the recurrent group compared to the non-recurrent group, which was consistent with the study by Wong JC et al. [17]. The expression of *TIMP* was up-regulated in the recurrent group. Similarly, Fong KM et al. [19] reported higher levels of *TIMP1* RNA in adenocarcinomas, which are relatively aggressive non-small cell lung cancers, and observed a striking association between these high levels and an adverse outcome. Holten-Andersen et al. [45] showed that high preoperative plasma levels of *TIMP1* are associated with short survival in CRC patients. Therefore, instead of inhibiting metastasis, *TIMP* may play a role in determining patient survival, possibly by promoting growth or another function.

## Conclusions

In summary, the molecular signature we identified in this study is closely associated with the clinical outcome of stage II/III CRC patients. This classifier is based on the mRNA expression levels of a set of 31 genes and can be used in a standardized assay for the validation of individual patients in a prospective study.
